# Identifying the key components of social support for patients living with type 2 diabetes: A protocol for a systematic review and meta-analysis of type 2 diabetes social support interventions

**DOI:** 10.1371/journal.pone.0306709

**Published:** 2024-08-01

**Authors:** Reda Madroumi, Lisa Newson, Frederick Kanayo Umeh, Helen Poole, Andrew Jones

**Affiliations:** Faculty of Health, School of Psychology, Liverpool John Moores University, Liverpool, United Kingdom; University of Benghazi, LIBYA

## Abstract

Type 2 diabetes (T2D) is a disease that impacts a huge portion of the world’s population. The number of T2D cases is expected to keep rising during the next decade. Committing to the treatment to manage this condition makes participants feel a burden of emotions making them require emotional support from caregivers or close ones. Support from family or caregivers can help improve glycaemia control, medication adherence, and T2D self-management. However, little is known about what aspects of social support effectively improve patients’ T2D self-management outcomes. The aim of this systematic review and meta-analysis is to identify the effective components of social support that can help participants improve their T2D self-management. **Methods:** The protocol of this review was developed based on the Preferred Reporting Items for Systematic Reviews and Meta-analyses (PRISMA) guidelines. The PRISMA recommendations were applied to develop a search strategy in collaboration with a team of academics to identify relevant T2D social support interventions via healthcare and psychology databases, Medline, Web of Science, ProQuest, CINHAL. **Discussion:** This review will provide an overview of what intervention social support components have a significant impact on T2D glycaemia control. These findings will inform future T2D interventions on what social support components could be used to encourage better diabetes self-management and glycemic control.

## Background

### What is type 2 diabetes

Type 2 Diabetes (T2D) is a metabolic disorder that causes glucose to build up in the blood [1]. This can be due to insufficient insulin production (Pancreas not producing enough insulin) or having insulin-resistant cells where insulin receptors fail to absorb glucose into body cells [1]. Treatment in T2D is aimed at maintaining blood sugar levels within a specific range, which also prevents future health complications [2]. Since treatment is mainly managed by patients and their families, following a complex set of behavioural actions is required to care for T2D [2]. A preferred terminology for this is self-management, and it simply refers to a set of skilled behaviours to manage one’s illness [3]. This includes healthy eating, being physically active, monitoring blood sugar, compliance with medications and risk-reduction behaviours [2].

### Type 2 diabetes prevalence and risk factors

The number of T2D cases increased from 108 million in 1980 to 422 million in 2014 [4]. According to the International Diabetes Federation (IDF), the global prevalence of type 2 diabetes in adults was 536.6 million (10.5%) in 2021 and is expected to rise to 783.2 million (12.2%) by 2045 [5].

The risk factors of T2D are associated with, for example, age, obesity, and an unhealthy diet [6]. Moreover, physical and social environments are major influences on diet and physical activity, affecting economic, psychological, and cultural factors [7]. Furthermore, ethnicity is also strongly associated with T2D prevalence since people of South Asian, African-Caribbean, Chinese or black African get T2D up to a decade earlier than White Europeans [8].

It is worth noting that the COVID-19 pandemic heavily affected diabetes patients in terms of physical and dietary practices [9]. This has been linked to fluctuating and elevated glucose levels in T2D patients which can be a sign of a poor prognosis and be challenging to manage [10]. The lack of medical support and the circumstances that T2D patients were exposed during the pandemic (e.g., spending most of the time at home with family) served as a reminder of the role of social support in improving T2D management outcomes [11].

Thus, multi-sectoral solutions involving society and T2D patients’ close family members are needed to tackle the numerous T2D health complications and its economic burden on affected citizens and governments [12].

### Social support in type 2 diabetes

In terms of T2D management, social support can be defined as using social resources for health management behaviours [13]. Social support can be related to proximal factors (e.g., friends and family) or distal factors (e.g., neighbourhood or community). Rajati et al. [14] mentioned that social support in T2D is considered an important element in improving patients’ mental health by gaining a sense of belonging in their social network. Additionally, social support from family and friends significantly influences patients’ chronic illness self-management [15].

### The four categories of social support

There are four diabetes-related social support components associated with type 2 diabetes: Informational, emotional, tangible, and appraisal. Informational support can refer to teaching patients about their own condition or learning from the experiences of others [16,17]. Emotional support entails reassuring patients and expressing empathy and caring gestures [17]. Tangible support focuses on providing patients with support materials to assist them with self-management [17]. Finally, the appropriateness of actions or statements that strengthen the patient’s beliefs and behaviours is validated by appraisal support [18].

### Type 2 diabetes social support interventions

Recent research has shown that involving family members in health education for T2D patients can improve glycaemic control, medication adherence and self-management [19–22]. Furthermore, high levels of social support were found to help with the impacts of T2D on health and reduce distress for patients [23,24]. Thus, social support could also be beneficial in diagnosis acceptance and emotional adjustment [25].

A systematic review conducted by van Dam et al. [26] revealed that the effect of social support interventions in T2D had not been extensively researched. Moreover, the authors mentioned that their findings did not reveal which aspects of social support are most effective for T2D self-management. Strom and Egede [27] reached a similar conclusion, stating that there is a need for a better understanding of the functions of social support.

### Aims of this review

In order to address the gap of better understanding of which aspects are most effective in T2D management and its functions of social support [26,27]. This review will aim to identify social support components in type 2 diabetes interventions. This will be accomplished by categorizing intervention procedures into the four social support components listed above (informational, tangible, emotional, and appraisal). This will enable us to compare interventions and determine which aspects of social support are most effective.

## Methods and analysis

### Aims and questions

This review aims to identify the effective intervention social support components that help participants improve their type 2 diabetes self-management and glycemic control. These interventions can include T2D patients and their family members, caregivers, or peers.

Three questions will be posed:

1. What T2D social support components have been used in previous interventions since 1990?
2. Which of these components of social support had a significant impact on patients’ glycemic control?
3. Is there a difference between the components used in interventions with significant vs. non-significant effect on biological indicators?

### Study design and eligibility criteria

Any peer-reviewed primary studies which assessed interventions targeting the use of social, peer or family support to improve T2D self-management. Only studies in English will be included. Studies published before 1990 will be excluded to ensure the inclusion of more relevant literature. For more representativeness, only male or female studies will be excluded. Qualitative design studies and the ones with no biological indicators will be excluded as they will not allow for comparing the effect of social support on glycemic control.

### Participants

Patients living with type 2 diabetes, aged 18 or more will be included. Patients with other types of diabetes (e.g., type 1 or gestational diabetes) will be excluded from the review. Moreover, the review will exclude any patients with a psychological condition (e.g. Alzheimer’s, Schizophrenia, depression, or post-traumatic disorder) or comorbidity. The reason behind this was to avoid any confounding factors that may affect the diabetes indicators.

### Interventions

Type 2 diabetes interventions targeting to improve or explore the effect of social support on T2D self-management. The interventions can include any family members, carers, or peers.

### Comparison

This systematic review and meta-analysis will compare intervention groups with control groups in studies identified through our search. The definitions of these groups are as follows:

- Intervention Group: Any groups that includes participants who received targeted social support interventions even if accompanied by regular medical treatment and clinical management as prescribed by healthcare providers.
- Comparison Group: Any groups who comprise of individuals who received standard clinical treatment without the additional layer of social support interventions. It also includes any group that serves as a baseline to evaluate the effectiveness of social support interventions when added to regular clinical treatment.

To provide a comprehensive analysis, this review will conduct both between-group (intervention vs. control) and within-group comparisons, assessing changes from baseline at various follow-up intervals.

### Outcomes

Studies that include an assessment of HbA1c as it gives a direct indication of glycemic improvements.

### Types of studies

This systematic review and meta-analysis will be limited to RCT’s, quasi-experiment and mixed method studies.

### Search strategy

The search method will be developed in collaboration with a team of academics that have substantial experience building search strategies using relevant psychology and healthcare databases. The search will take place in December2023. The following electronic databases will be searched: Medline, Web of Science, Pro Quest, CINHAL.

Interventions of adult populations with type 2 diabetes published between 1990 and 2023 will be used to filter the results. The choice of the year 1990 as the starting point is strategically made to cover the significant advancements in diabetes management and social support strategies that have emerged since then. This approach ensures a comprehensive and contemporary overview of intervention strategies in the field. This will be mapped across all databases listed above. The reference lists of the publications designated as appropriate were carefully searched for other potential interventions.

### Data management

Following database searching, studies will be populated into Rayyan AI [28], which will manage study selection and data extraction. After study de-duplication through Rayyan, double screening will be employed, in which two reviewers independently assess titles and abstracts. RAYYAN AI [28] will be used as a tool to review the full texts of retrieved articles selected from database. Disagreements should be debated until a consensus is established.

We will contact the study authors to request incompletely reported data in included studies. We will conduct analyses using available data if no response is received within 14 days.

### Assessments of methodological quality

Since this systematic review includes quantitative, and mixed-methods research, the Mixed Methods Appraisal Tool (MMAT) [29] was utilized to assess the risk of bias. The MMAT provides a checklist that includes two screening questions that check if the study is empirical allowing the reviewers to decide whether they can use the MMAT as an appraisal tool [29]. The MMAT also offers appraisal questions for each study design allowing for fair appraisal and comparison between the different designs. This tool also requires at least two reviewers to be involved in the appraisal process [29]. This review will do as well by involving one researcher and 3 academics to independently appraise the studies selected. The MMAT encourages us to provide a detailed justification of each criterion scoring to have a clearer idea of the overall quality of each study appraised.

### Data summary

A summary of the data results will be presented in conformance with the PRISMA checklist requirements [30]. This will include a summary table that cites each study’s characteristics. The table will be divided into three main sections: *Study ID* (Authors and year of publication), *Population* (Country, Intervention context, Sample size, Age, and gender), and *Outcomes* (Follow-up and HbA1c results). Another table will be included to support giving more information about which categories of social support used in each intervention it will contain the following: *Intervention social support categories* (Tangible, Informational, Emotional and Appraisal support).

### Study limitations

Not including qualitative studies in this systematic review can be a limitation as deeper insights about what might be effective in social support might not be considered. Nevertheless, this review still considers including mixed methods studies. This will allow for measuring the interventions’ impact on T2D self-management and comparing them to other trials. Furthermore, qualitative insights can be looked at to understand why specific components were effective or failed in enhancing self-management and glycemic control.

### Quantitative synthesis

The studies that will show a significant clinical, methodological, and statistical heterogeneity will be synthesized quantitatively based on their follow-up periods and type of intervention (e.g., family, peers, or couples’ interventions). To accurately classify the interventions into one of the four social support categories—informational, tangible, emotional, and appraisal—the research team will systematically assess each intervention’s protocol. This assessment will be guided by a predefined set of criteria, which aligns with the definitions provided by [16–18]. This structured approach ensures that each intervention is categorized based on the type of social support it primarily offers, facilitating a clear and consistent analysis.

### Measure of effect sizes

Mean difference will be used as the measure of effect, as the outcome (HbA1c) will be the same across each study. In between-subject designs, the mean difference will be calculated as the difference between groups at post-intervention and follow-up periods. For any within-subject contrasts, the mean difference will be the difference between pre- and post-intervention and any follow-ups.

### Assessment of heterogeneity

I2 index will be used to quantify the heterogeneity. It will allow us to estimate the percentage of variability in studies’ results that is due real differences rather than chance [31].

### Quantitative data synthesis

Quantitative synthesis will be conducted using a multilevel, random effects meta-analysis in R, utilizing the ‘metafor’ package. A multilevel meta-analysis will be conducted to accommodate studies that provide multiple effect sizes for the model, arising from multiple follow-up periods [32].

### Publication bias

The risk of publication bias will be evaluated using Trim and Fill [33], Egger’s test and GOSH approaches [34,35].

### Confidence in cumulative evidence

The results will be interpreted using the Joanna Briggs Institute (JBI) assessment tool [36]. This tool provides a systematic methodology for assessing the robustness and reliability of research [36]. It closely examines essential aspects of the study design, such as randomisation, blinding, data collection methods, and analysis techniques, ensuring that systematic reviews and meta-analyses are grounded in methodologically solid studies [36].

## Discussion

Social support has been linked to better type 2 diabetes self-management as well as improved patient wellbeing and quality of life [14,15]. However, it has been stated that aspects of social support are not understood, and that more research is needed to determine which functions are most effective [26,27]. Type 2 diabetes was classified into four types of support (emotional, educational, informational, and appraisal) [16–18]. Using these four categories and evaluating how they were used in previous social support interventions can help identify which aspects are important for improving type 2 diabetes self-management. This will help to inform future research and point to best practices for designing social support interventions aimed at improving type 2 diabetes self-management.
